# Impact of Blood Herpes Simplex Virus Polymerase Chain Reaction Testing in Neonatal Management of Herpes Simplex Virus Infection: An Institutional Experience

**DOI:** 10.7759/cureus.102996

**Published:** 2026-02-04

**Authors:** Ana M Alvarez, Melanie Vega, Frances Valencia-Shelton, Mobeen H Rathore

**Affiliations:** 1 Pediatric Infectious Diseases, University of Florida College of Medicine – Jacksonville, Jacksonville, USA; 2 Infectious Diseases, Baptist Medical Center Jacksonville, Jacksonville, USA

**Keywords:** herpes simplex virus, hsv pcr, hsv testing in blood, neonatal hsv diagnosis, neonatal hsv infections

## Abstract

Background: The evaluation of neonatal herpes simplex virus (HSV) includes HSV culture and/or polymerase chain reaction (PCR) of different surfaces, cerebrospinal fluid (CSF), and blood. However, the impact of blood HSV PCR for the management of neonatal HSV infections has not been well-established.

Objective: To evaluate the impact of blood HSV PCR results in the management (i.e., clinical decision-making regarding treatment) of neonatal HSV infections at our institution, where this test is performed at an outside facility.

Methods: We retrospectively reviewed the medical records of all neonates tested for HSV DNA in blood from January 1, 2018, to July 31, 2023, at our children’s hospital.

Results: A total of 149 medical records were analyzed, of which seven had positive blood HSV PCR. These seven patients and five of 142 patients with negative blood HSV PCR had a positive HSV PCR result from another site, and they were managed according to those results. The mean turnaround time (TAT) for the results of blood HSV PCR was three and a half days for positive results and four days for negative results. In most patients with negative PCR from other sites, including CSF, acyclovir was discontinued before the blood HSV PCR results were back. Acyclovir was not discontinued until the results were available in only 12 of 134 neonates who had all negative results and available information about management. Statistical analysis showed that blood HSV PCR did not significantly impact the management of these neonates.

Conclusions: Our study does not support the use of blood HSV PCR in the evaluation of neonatal HSV infection specifically in institutions where the test is not performed in-house, because the test results are not available in a timely fashion to have an impact on the management of these neonates.

## Introduction

Herpes simplex virus (HSV) infections in neonates are rare but can cause devastating disease. Although neonatal HSV infection has a broad clinical spectrum, it remains a major cause of neonatal morbidity and mortality [1,2]. The fatality rate of neonatal HSV can be as high as 30% for disseminated infection, even with the use of high-dose acyclovir [1]. These infections can manifest in three forms: skin, eye, and mouth (SEM); central nervous system (CNS); and disseminated disease. Testing for these infections, as outlined by the American Academy of Pediatrics (AAP), includes viral cultures and/or HSV PCR testing of different sites such as body surfaces (conjunctiva, nasopharynx and anus), cerebrospinal fluid (CSF) and blood [3].

Blood HSV PCR has been found to be positive in some infants with SEM, CNS, and disseminated disease, as well as in healthy children with primary gingivostomatitis and in healthy infants from four to 36 months of age with and without eczema presenting with vesicles with or without fever [4]. The American Academy of Pediatrics states that, because positive blood HSV PCR does not correlate with the type of disease, it should not be used to determine the extent of disease or the length of treatment [3]. Additionally, blood HSV PCR can be positive for weeks even in the presence of adequate antiviral therapy, making it of unclear significance and not useful for monitoring the response to therapy [3,5]. For these reasons, the usefulness of HSV PCR testing in blood has been questioned [6].

It is also notable that prolonged testing turnaround time (TAT) for the results of blood HSV PCR, as when tests are not performed in-house, may affect the ability of the results to be used for the management of these patients. Considering diagnostic stewardship, the value of testing with longer TAT when performed in an outside laboratory should be questioned. At our institution, it is a standard practice to follow AAP recommendations regarding diagnostic tests to be sent in neonates with suspected HSV. These recommendations have included HSV PCR in blood since at least 2015. In our center, HSV PCR in CSF and different surfaces is performed in-house while the HSV PCR in blood is performed at an outside facility. The objective of this study was to evaluate the impact of blood HSV PCR results in the management (i.e. clinical decision-making regarding treatment) of neonatal HSV infections at our institution.

## Materials and methods

We performed a retrospective review of the electronic medical records (EMR) of all neonates (less than four weeks of age) who underwent blood HSV PCR testing from January 1, 2018, to July 31, 2023, at our children’s hospital (276-bed freestanding children’s hospital in the southeastern US). This information was obtained from the laboratory database for all orders of blood HSV PCR in the study population at our hospital. Data were de-identified.

The study was reviewed by the hospital’s Institutional Review Board (IRB), and it was determined to be exempt from IRB review per 45 Code of Federal Regulation (CFR) 46.104(d)(4). Information collected from the EMR included age, sex, admission diagnosis, final diagnosis, type of HSV disease, results of all HSV PCR tests performed (CSF, surface swabs including skin, conjunctival, rectal, and oro/nasopharynx, and blood), liver enzymes, prescribed antimicrobials, and their duration.

The TAT for blood HSV PCR was collected for both positive and negative results. Based on a review of providers’ free-text notes by a single investigator with a basic coding system with predefined criteria, the impact of blood HSV PCR in the management of HSV infection was determined by identifying whether the results (positive or negative) of blood HSV PCR affected the management. We defined management as the use of acyclovir (i.e., if acyclovir was started, continued, or discontinued based on the results). When the reason for continuing or discontinuing acyclovir was not specified in the provider’s note, we counted these charts as “information about management missing,” and they were excluded from the analysis of the impact of blood HSV PCR results on management.

Statistical analysis was done using SPSS version 30. We used Tukey’s hinges to calculate the median, 25th, and 75th quartiles for age. Exact (Clopper-Pearson) 95% confidence intervals were calculated for the marginal positivity rates of each test and for the discordant proportions to quantify the precision of these estimates. We used McNemar’s test to determine if there was a statistically significant difference in performance between the blood HSV PCR and HSV PCR in other sites, and to determine if the blood HSV PCR impacted the management significantly. Because of the small number of discordant pairs, the two-tailed p-value was calculated using the exact binomial test for paired proportions.

## Results

**Figure 1:**
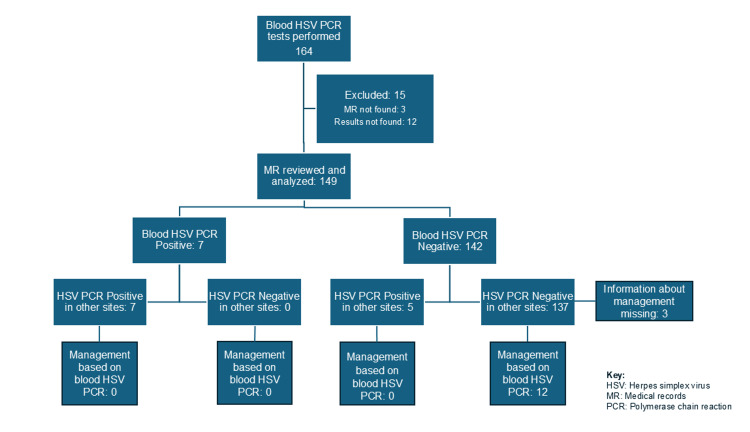
Flow chart of neonates who had a blood HSV PCR test performed from January 1, 2018 to July 31, 2023

A total of 164 blood HSV PCR tests were performed in neonates within the specified timeframe at our institution. These tests were performed at a commercial laboratory using a real-time PCR assay with a limit of detection between 60 to 150 copies/ml. Three charts were not found, and 12 charts did not have documented results. The remaining 149 charts were analyzed (Figure 1).

The median age (25th and 75th interquartile range) for the cohort was six days (3, 14). The median ages of neonates with positive and negative blood HSV PCR results were 16 (11, 22) and five (2, 14) days, respectively. The median ages of neonates with positive and negative HSV PCR in other sites were 12.5 (8.5, 21) and five (2, 14) days, respectively. When we compared the age differences using the independent-samples Mann-Whitney U test, the neonates with negative results were younger than the ones with positive results, and these differences were statistically significant with a p-value of 0.005 for blood and 0.011 for other sites. The mean turnaround time (TAT) for the results of blood HSV PCR was three and a half days (range: 3-4) for positive results and four days (range: 3-6) for negative results.

Among 149 paired samples, the blood PCR was positive in seven of 149 samples (4.7%, 95% CI 1.9-9.5%), whereas other sites tested positive in 12 of 149 samples (8.1%, 95% CI 4.2-13.7%). All patients with positive blood HSV results had a positive HSV PCR result from another site (four in CSF, one in CSF and skin, and two in skin). For discordant pairs, five (3.4%, 95% CI 1.1-7.7%) were positive in other sites but negative on blood PCR, and 0 (0%, 95% CI 0-2.5%) were positive on blood PCR and negative at other sites. Of all patients with positive HSV PCR results in the blood, three had disseminated disease, three had CNS disease, and one had SEM disease. Of the three patients with disseminated disease, two tested positive in CSF and one on a skin lesion. Five (4%) of the 142 with negative blood HSV PCR had a positive HSV PCR from another site and were treated according to their site of infection (four CNS and one SEM). Patients with HSV meningoencephalitis or disseminated disease were treated for 21 days. Patients with SEM without any signs of systemic infection were treated for 14 days.

Specific information about management was missing in three of 149 neonates. All three had negative HSV PCR results in blood and other sites. They were excluded from the analysis of the impact of blood HSV PCR results on management. In 122 (91%) of the 134 neonates with negative HSV PCR testing and known management, acyclovir was stopped when HSV PCR results from other sites were negative (in less than 36 hours) and before the negative blood HSV PCR results were reported. The decision to stop acyclovir was made after the results of blood HSV PCR were reported negative in 12 (9%) of 134 patients. The main reasons why acyclovir was continued until blood HSV PCR results returned in these 12 patients were provider-dependent and are listed in Table 1. Four of the 12 neonates were seven days of age or younger. Notably, none of the 12 patients had a final diagnosis of HSV infection.

**Table 1:** Reasons for Continuing Acyclovir While Waiting for Blood HSV PCR Results HSV: herpes simplex virus, CSF: cerebrospinal fluid

Reasons	Number of patients
Seizures that were not improving	4
Continued apnea	3
CSF pleocytosis and increased protein	1
Persistent fever and transaminitis	1
Persistent seizures and transaminitis	1
Respiratory distress	1
Worsening transaminitis	1

Statistical analysis using the McNemar’s exact binomial test for paired proportions showed that the blood HSV PCR testing did not perform significantly differently from the other tests, such as HSV PCR in CSF or HSV PCR surface swabs, with a two-tailed p-value of 0.063 (Table 2). However, the small number of discordant pairs and the very low prevalence of positive blood HSV PCR results limit the statistical power to detect modest differences between the two types of assays. As a result, the possibility of a type II error cannot be excluded, and the borderline p‑value should be interpreted in the context of these sample size constraints. In regard to impact on management, blood HSV PCR testing did not significantly impact the management of these neonates with a two-tailed p of 0.359 (Table 3). However, given the very low number of positive blood HSV PCR results, the study has limited power to determine true lack of impact.

**Table 2:** Performance of Blood HSV PCR Compared to Other HSV PCR Testing Sites Blood HSV PCR did not perform significantly differently than HSV PCR in other sites. P-value was calculated using McNemar’s exact binomial test for paired proportions. HSV: herpes simplex virus; PCR: polymerase chain reaction.

Variable		HSV Testing in Other Sites		p-value (2-tailed)
		Negative	Positive	
Blood HSV PCR	Negative	137	5	0.063
	Positive	0	7	

**Table 3:** Impact of Blood HSV PCR Results on the Management of Neonates Evaluated for HSV Infection Blood HSV PCR results did not significantly affect the management of neonates evaluated for HSV infection. P-value was calculated using McNemar’s exact binomial test for paired proportions. HSV: herpes simplex virus; PCR: polymerase chain reaction.

Variable		Management		p-value (2-tailed)
		Not Affected	Affected	
Blood HSV PCR	Negative	127	12	0.359
	Positive	7	0	

## Discussion

Blood HSV PCR testing at our institution did not have a significant impact on the management of neonatal HSV infection. The current literature contains only a limited number of targeted studies on the usefulness of blood HSV PCR in neonates. One study of prospectively collected plasma for HSV PCR in 47 neonates showed that 14 (78%) of 18 neonates with SEM, 7 (64%) of 11 neonates with CNS, and 18 (100%) of 18 neonates with disseminated disease had HSV viremia [5].

However, another study that aimed to determine the utility of blood HSV PCR in neonatal HSV infection found HSV DNA detectable in blood in 13 (50%) of 27 neonates with HSV infection: 4 (100%) of 4 patients with disseminated disease, 4 (57%) of 7 patients with CNS disease, and only 4 (28%) of 14 patients with SEM disease [7]. Interestingly, in this last study, a patient with a positive HSV PCR in the blood who tested negative on repeat testing was diagnosed with enterovirus meningitis and recovered without treatment for neonatal HSV. This was considered a false positive result [7]. A recent study compared the value of sent-out blood HSV PCR versus the in-house test in patients aged <60 days who underwent neonatal HSV evaluations [8]. The study included 131 patients who underwent sent-out blood HSV PCR and 128 patients who underwent in-house tests. In the sent-out group, only one patient had positive blood HSV PCR results; this patient also tested positive on surface swabs, had transaminitis, and was diagnosed with disseminated disease. In the in-house group, two patients had positive blood HSV PCR results. One of them tested positive in CSF and surface swabs, had transaminitis, and was diagnosed with disseminated HSV. The other patient only had positive HSV PCR results in the blood and was diagnosed with disseminated disease. However, this last patient did not have transaminitis or thrombocytopenia, which made the disseminated disease questionable. Thus, two of the three neonates with disseminated disease had positive PCR results at another site, transaminitis, and thrombocytopenia, and it is likely that they would have been treated regardless of the blood HSV PCR result. Blood HSV PCR results were negative in another four patients with other types of neonatal HSV infections (three SEM, one CNS) [8].

In the same study, the authors compared the influence of in-house versus sent-out blood HSV PCR on turnaround time (TAT), acyclovir duration, and length of hospital stay [8]. They found that the in-house test optimized these three parameters, as shown previously [9]. The authors also noted that in their sent-out cohort, the median TAT was longer than the median acyclovir duration [8]. This suggests that when the test was not available in-house, many clinicians did not wait for the blood HSV PCR results before discontinuing acyclovir, which is similar to the findings in our study. Ahmat et al. evaluated the impact of institutional guidelines for HSV screening and initiation of empiric acyclovir treatment in neonates in their ED. The authors found it useful, but they noted that PCR testing on both CSF and blood was performed at their in-house virology lab with a standard TAT of less than 36 hours [10]. In a review of the diagnosis and management of neonatal herpes simplex infection in the ED, other investigators noted that the pathways created for evaluating these patients will depend on both laboratory TAT and the local incidence of HSV infections [11]. These studies highlight the impact of laboratory testing location and the TAT of results on how the results of HSV PCR can be used in clinical decision-making.

In our study, blood HSV PCR had no impact on management decisions in neonates with positive results. All neonates with positive blood HSV PCR results had positive HSV results at other sites and were treated according to their clinical presentation, regardless of the results in blood. The McNemar test did not demonstrate a statistically significant difference between blood PCR and the composite other sites in our study. However, the discordant results may offer some clinical insight. Cases that were blood‑negative but positive at other sites (CSF, skin culture, or lesion PCR) may reflect a higher sensitivity of site‑specific sampling for localized HSV infection. This puts in question the need to obtain blood HSV PCR when CNS and SEM disease is suspected. On the other hand, the absence of blood‑positive/other‑site‑negative cases in our cohort suggests that blood PCR alone is insufficient to exclude infection when the clinical suspicion is high. These patterns reinforce the importance of obtaining samples from the most clinically relevant anatomic sites and suggest that blood PCR and other‑site testing provide complementary diagnostic information. Additionally, the blood HSV PCR results had no significant impact on the decision to discontinue treatment in 91% of the patients with negative blood HSV PCR. It only had an impact on 9% of neonates with negative blood HSV PCR results. However, the very low number of positive blood HSV PCR results limits the statistical power of these results.

Regarding the age of neonates at presentation, in our study, the median age of neonates with positive blood HSV PCR (16 days) was significantly higher than the median age of neonates with negative HSV PCR results (5 days). This difference was statistically significant. This age is slightly higher than the age of onset of symptoms typically seen in neonatal disseminated disease and SEM disease (1st and 2nd week of life), but it aligns with the typical age of onset in CNS disease (2nd and 3rd weeks of life) [3]. This could potentially represent patients presenting late in the course of the disease. The median age of the total cohort was 6 days, which indicates that there is a high index of suspicion in our institution when babies present with symptoms in the first week of life. Additionally, it is possible that the decision to wait for the blood HSV PCR results to discontinue acyclovir was affected by the age of the neonates at presentation. However, only 4 of 12 neonates who continued receiving acyclovir while waiting for the result of the blood HSV PCR were 7 days old or younger. This suggests that the decision to continue acyclovir was more related to the clinical signs and symptoms than the age of the neonate.

Ours is one of the few studies that looked at the TAT of blood HSV PCR results for inpatient cases in the context of not having that test in-house and analyzed its impact on the management of these patients. Our findings suggest that TAT is a key driver of management decisions. In institutions with in-house blood HSV PCR testing, having the results in a timely fashion can be helpful and complementary to the HSV PCR in other sites. However, our study reflects the reality of many hospitals that do not have in-house testing for blood HSV PCR and it questions the utility of this send-out test, especially when considering diagnostic stewardship. In 100% of our patients with positive results and 91% of our patients with negative results, the test had no impact on the management of these patients, mostly due to long TAT. On the other hand, keeping the patients in the hospital receiving acyclovir while waiting for the results would have meant 2-4 extra days of antiviral exposure and hospitalization with all the resulting economic and emotional costs. In institutions with quick TAT for this test, blood HSV PCR testing offers some advantages to reduce exposure to acyclovir and duration of hospitalization, especially in cases of suspected disseminated infection when other tests are negative.

The limitations of our study include its retrospective nature, single-center experience, the low number of neonates with positive HSV PCR results in blood, which makes the study statistically underpowered, and the lack of a two-independent-reviewer system to perform the review of the providers’ notes. For this last point, we minimized subjectivity by using a basic coding system with predefined criteria. Even with these limitations, this study reflects the reality of many hospitals in the US with limited capabilities to perform in-house blood HSV PCR and it questions the usefulness of this test when the TAT is longer than 36-48 hours.

## Conclusions

In conclusion, although there may be advantages to performing in-house blood HSV PCR testing for the evaluation of neonatal herpes, our study does not support the use of blood HSV PCR in the evaluation of neonatal HSV infection specifically in institutions where the test is not performed in-house, because the test results are not available in a timely fashion to have an impact on the management of these neonates. Additional larger studies that specifically examine the effects of TAT of blood HSV PCR in the management of neonatal HSV (e.g. acyclovir use, length of stay, safety endpoints, etc.) are needed to confirm our findings and to optimize resource utilization while maintaining patient safety.
